# Contribution of the paternal histone epigenome to the preimplantation embryo

**DOI:** 10.3389/fcell.2024.1476312

**Published:** 2024-11-12

**Authors:** Ashton R. Dodd, Lacey J. Luense

**Affiliations:** ^1^ Department of Animal Science, Texas A&M University, College Station, TX, United States; ^2^ Genetics and Genomics Interdisciplinary Program, Texas A&M University, College Station, TX, United States

**Keywords:** epigenetics, spermatogenesis, histone modifications, embryo development, sperm epigenome

## Abstract

The paternal germline contains a plethora of information that extends beyond DNA. Packaged within the sperm cell is a wealth of epigenetic information, including DNA methylation, small RNAs, and chromatin associated histone proteins and their covalently attached post-translational modifications. Paternal chromatin is particularly unique, as during the process of spermatogenesis, nearly all histones are evicted from the genome with only a small percentage retained in the mature sperm cell. This paternal epigenetic information is encoded into chromatin during spermatogenesis and is delivered to the oocyte upon fertilization. The exact role of these paternally contributed histones to the embryo remains to be fully understood, however recent studies support the hypothesis that retained sperm histones act as a mechanism to poise genes for early embryonic gene activation. Evidence from multiple mammalian species suggests sperm histones are present at loci that are important for preimplantation embryo chromatin dynamics and transcriptional regulation. Furthermore, abnormal sperm histone epigenomes result in infertility, poor embryogenesis, and offspring development. This mini-review describes recent advances in the field of paternal histone epigenetics and their potential roles in preimplantation embryo development.

## Introduction – advancements in epigenetics

Our knowledge of the genome has advanced significantly during the twenty-first century. Advances in proteomics, structural biology, microscopy, and next-generation sequencing technologies have provided unprecedented access to investigating chromatin structure and function. One particularly exciting area of advancement and growth is the field of epigenetics. The study of epigenetics encompasses multiple heritable facets affecting gene expression without altering the underlying DNA sequence. This includes the addition of chemical modifications to DNA and chromatin, which ultimately alters the structure and function of DNA and gene expression. These epigenetic modifications occur in all cell types and are a major regulator of cell differentiation, organismal development, and normal cellular function (Allis and Jenuwein, 2016). The male germline, beginning with immature germ cells and continuing to the mature sperm cell, is uniquely regulated by multiple epigenetic mechanisms. Furthermore, epigenetic regulation is a critical mechanism that mediates key developmental time points and is particularly susceptible to alterations from endogenous mutations or outside environmental perturbances, thus underscoring the importance of understanding the role the epigenome plays in the male germline (Radford, 2018).

Several epigenetic mechanisms are encoded in the male germ cell during the process of spermatogenesis and are passed to the embryo upon fertilization. These mechanisms regulate DNA and chromatin structure, as well as transmit noncoding RNAs. Chromatin consists of DNA and associated proteins and is a key mediator of gene regulation. The primary structure of chromatin begins with the nucleosome, composed of two copies each of four core histones, H2A, H2B, H3, and H4 (Kornberg, 1974), with approximately 147bp of DNA wrapped around the histone octamer. Histone proteins contain an N-amino tail that extrudes from the nucleosome and can be decorated with a variety of post-translational modifications (PTMs) that include, but are not limited to, acetylation, phosphorylation, ubiquitination, and methylation (Zhao and Garcia, 2015). These covalently attached PTMs impact numerous nuclear and cellular processes including transcription, DNA repair, and cell cycle progression (Ehrenhofer-Murray, 2004). Histone PTMs allow for relaxation or condensation of chromatin and serve as mediators for the binding of transcription factors and other proteins that regulate chromatin structure and function. For example, chromatin accessibility, or the controlled ability of transcription factors and other molecules to gain and maintain physical access to chromatin, is frequently linked to acetylated histone PTMs (Mansisidor and Risca, 2022). Alternatively, chromatin condensation is typically mediated through histone methylation and results in restriction of chromatin accessibility. Higher order chromatin structure, or the 3D genome, is an additional epigenetic mechanism that regulates gene accessibility and cellular function, particularly during development (Lupiáñez et al., 2015). Chromatin structure is particularly unique in male germ cells, playing key roles in germ cell maturation, while further transmitting critical epigenetic information to the embryo.

**FIGURE 1:**
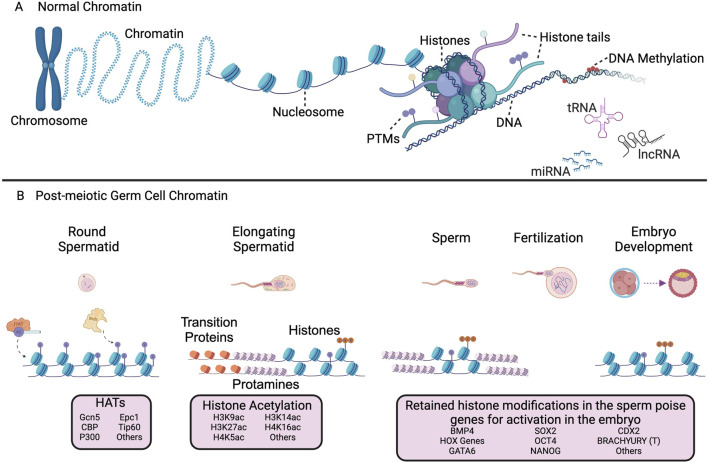
Schematic representation of the epigenetic mechanisms in normal and post-meiotic germ cell chromatin. **(A)** Chromatin structure of somatic and non-spermatid cells and additional mechanisms of epigenetic regulation. Multiple epigenetic mechanisms contribute to the regulation of chromatin dynamics, including histone post-translational modifications (PTMs), DNA methylation, and non-coding RNAs. **(B)** Chromatin function and regulation in post-meiotic germ cells, mature sperm, and the preimplantation embryo. Histone acetyltransferases (HATs) act as ‘writers’ through the addition of acetyl groups to lysine residues of histone proteins. The BET family member BRDT is one example of a ‘reader’ protein that binds acetylated histones for eviction and subsequent replacement by transition proteins and ultimately protamines. Retained histones in mature sperm are transmitted to the early embryo following fertilization and poise chromatin for subsequent regulation. Genes with retained histones were identified in the following publications (Hammoud et al., 2009; Brykczynska et al., 2010).The HATs, ncRNAs, embryonic genes, and PTMs in this figure are not a comprehensive list. Image created with BioRender.com.

While the primary focus of this mini-review is the regulation of the histone epigenome in sperm, several other key epigenetic mechanisms exist and are critical for sperm function (Figure 1A). DNA methylation occurs when the fifth carbon of cytosine bases are modified with the covalent addition of a methyl group (Figure 1A). DNA methylation typically occurs at gene promoters or CpG islands to repress gene transcription and is essential in the regulation of a multitude of cellular processes (Greenberg and Bourc’his, 2019). DNA methylation is an essential component of reproductive epigenetics and early embryogenesis (Rivera and Ross, 2013). Noncoding RNAs (ncRNAs), including micro-RNAs (miRNA), transfer-RNAs (tRNAs), piwi-interacting RNAs (piRNAs), long non-coding RNAs (lncRNAs), and others, mediate epigenetic regulation by pairing with complementary RNA sequences to silence gene expression (Figure 1A) (Bartel, 2009; Guo et al., 2010). ncRNAs can be transmitted through cell division or through the germline to mediate inter- or transgenerational inheritance in multiple species (Conine et al., 2013; Zhang et al., 2019). A number of sperm borne ncRNAs, including miRNA and tRNA-fragments (tRFs), have been found to be transmitted to the embryo and regulate preimplantation embryo development (Sharma et al., 2016). Additionally, many of these epigenetic mechanisms act in concert, for example, miRNAs have been demonstrated to influence DNA methylation levels by regulating enzymes responsible for methylation activity, such as DNA methyltransferases (DNMTs) (Yao et al., 2019). Long-interacting RNAs (lincRNAs), have been demonstrated to interact with chromatin modifying enzymes, including the histone methyltransferase EZH2, to alter genomic histone methylation patterns (Ransohoff et al., 2018). Furthermore, metabolic disruptions caused by lifestyle and environmental factors can also impact the epigenetic regulation of the sperm and the subsequent development of the next-generation (Donkin and Barrès, 2018). These mechanisms have been studied in a variety of species, are active during spermatogenesis, and are paternally contributed to the embryo for subsequent regulation of development (Carrell and Hammoud, 2009; Carrell, 2012; Lismer and Kimmins, 2023).

## The sperm epigenome

The sperm epigenome is complex and contains a wealth of information that is essential in determining a viable gamete that insures male fertility and successful embryogenesis. Sperm chromatin exhibits a highly unique organization with nearly all histones replaced with small basic proteins termed protamines (Oliva, 2006; Carrell, 2012). This process of histone eviction occurs during the post-meiotic stage of spermatogenesis termed spermiogenesis and is critical for the journey through the female reproductive tract to reach the oocyte and initiate fertilization. During spermiogenesis, the haploid round spermatid undergoes chromatin compaction and condensation, cytoplasmic shedding, acrosome formation, and nuclear elongation (O’Donnell, 2014). The compaction of the nuclear material during this process aids in sperm motility, prevents oxidation and DNA damage, and primes the paternal genome to regulate gene expression in the embryo (Oliva, 2006). A key driver of histone eviction in spermatids is the hyperacetylation of histone lysine residues resulting in the relaxation of chromatin (Goudarzi et al., 2014). This allows for the eviction of histones which are replaced transiently by transition proteins (Tnp1 and Tnp2) prior to replacement by protamines (Figure 1B) (Meistrich et al., 2003). Protamines are arginine-rich proteins that allow for the formation of the toroidal chromatin structures that provide compaction of sperm chromatin. During protamination, 85%–99% of histones are evicted from chromatin and replaced by protamines, with only 1%–15% of histones (depending upon species) retained in the transcriptionally quiescent mature sperm (Figure 1B) (Ward and Coffey, 1991; Carrell, 2012). The proper establishment of the sperm epigenome is critical for normal fertility and development.

Protamines play an important role in compacting and protecting DNA. Protamine deficiency results in increased DNA damage, thus suggesting a protective function for protamines in maintaining DNA integrity (Aoki et al., 2006). In humans and other mammals, two protamine genes, Prm1 and Prm2 function together to initiate the proper condensation of sperm chromatin. Aberrations to the ratio of Prm1 and Prm2 have been linked to male infertility. Increased Prm1:Prm2 ratios are associated with decreased sperm penetration capacity and increased incidence of late spermiogenesis defects (Carrell and Liu, 2001). In non-human models, haploinsufficiency of Prm1 and Prm2 in mice results in infertility due to reduced motility, chromatin integrity, and morphology (Cho et al., 2001). Insufficient protamine incorporation is further indicative of increased retention of histones, as sperm from infertile men exhibited increased histone H2B and reduced Prm1:Prm2 (Zhang et al., 2006). Similar to histones, protamines can be modified with covalent post-translational modifications, including acetylation, methylation, and phosphorylation and are necessary for proper fertility and development (Brunner et al., 2014; Moritz et al., 2023; Schon et al., 2024). Mutation of lysine 49 of Prm1 in mice to an alanine residue prevented acetylation, resulting in decreased sperm motility and fertility defects (Moritz et al., 2023). In humans, men with male factor infertility exhibited increased phosphorylation of Prm1 (Schon et al., 2024). Thus, in addition to the increased susceptibility to DNA damage due to lack of protamines, aberrantly retained histone and associated PTMs may have additional functions that result in infertility.

Histone PTMs play a critical role during spermiogenesis in facilitating the removal of histones. Beginning in the late haploid round spermatid stage, histone lysine residues undergo hyperacetylation in a process that is vital to proper histone eviction (Marushige et al., 1976; Oliva et al., 1987). The strongest enrichment of acetylated lysines occurs on histone H4, with multiple residues including H4K5, H4K8, H4K12, H4K16 undergoing robust acetylation (Sonnack et al., 2002; Govin et al., 2006; Goudarzi et al., 2014; Luense et al., 2016). Hyperacetylated histones are also observed on other core histones including H3, H2A, and H2B (Brunner et al., 2014; Luense et al., 2016). Dynamic changes of individual histone PTMs have been identified through mass spectrometry and suggest temporal roles in regulating stage specific chromatin changes (Luense et al., 2016). For example, H4K12ac exhibits the highest enrichment in round spermatids, before decreasing in the more mature elongating stage, thus suggesting a role in initiation of histone eviction. In contrast, H4K5ac is low in round spermatids before increasing in the elongating and condensing stages of spermiogenesis, suggesting a role in a secondary wave of hyperacetylation during germ cell development (Luense et al., 2016).

Histone acetyltransferases (HATs) are enzymes responsible for acetylation of histone lysine residues (Marmorstein, 2001). Several different HATs are expressed during spermiogenesis in mammalian species and have been investigated for functional regulation of male germ cells (Figure 1B). Conditional knock-out of the HAT GCN5 (also known as KAT2A) in pre-meiotic male germ cells resulted in reduced histone acetylation, altered chromatin dynamics, increased histone retention and severe reproductive abnormalities including reduced testes size and sperm count, abnormal sperm morphology, and subfertility (Luense et al., 2019). Gcn5 primarily acetylates H3K9 and H3K14 (Grant et al., 1999; Bonnet et al., 2014) but has also been shown to acetylate H327ac and H4K5, K8, K12, and K16 (Kuo et al., 1996; Zhang et al., 1998). CBP and P300 are highly homologous HATs expressed during spermiogenesis that primarily acetylate H3K27 (Boussouar et al., 2014). Conditional double knockout of CBP and P300 in post-meiotic germ cells results in altered expression of metabolic remodeling genes normally expressed in elongating spermatids (Boussouar et al., 2014). However, conditional knockout of CBP and P300 did not result in altered histone acetylation during spermiogenesis, likely due to the redundant function of other HATs. EPC1 and TIP60, components of the NuA4 complex, acetylate lysine residues on histone H4. Knockout of EPC1 and TIP60 both demonstrated aberrations in hyperacetylation, and histone eviction required for normal elongating spermatid development, resulting in a reduction of elongating spermatids and mature spermatids (Dong et al., 2017). The role of HATs in the acetylation of histones during spermiogenesis are indispensable for proper chromatin organization and the production of mature sperm.

While HATs are the major ‘writers’ of histone acetylation, ‘reader’ proteins have been identified that bind acetylated histones in male germ cells. Bromodomain and extra-terminal domain (BET) family proteins including BRD2, BRD3, BRD4, and the testis specific BRDT, are expressed in mammalian spermatogenesis (Shang et al., 2004). These proteins include bromodomains, evolutionary conserved protein domains that bind acetylated histones. In mouse male germ cells, a tight temporospatial regulation of BET proteins are observed, suggesting stage specific regulation and binding of acetylated histones in the testis. BRD4 binds acetylated H3 and H4 histones in round spermatids, concomitant with acrosome formation (Bryant et al., 2015). BRDT binds acetylated histones and subsequently directs histone eviction and subsequent replacement by transition proteins (Gaucher et al., 2012). Mice with a truncated mutant BRDT allele produced sperm with abnormal morphology and motility (Shang et al., 2007). Small molecule bromodomain inhibitors, including JQ1 have been used to successfully block spermatogenesis and sperm production through targeting of BRDT and are being further investigated as potential modes of male contraceptives (Matzuk et al., 2012). The potential for pharmaceutical intervention to modify histone PTMs further underscores the complex regulatory systems, including the writers and erasers of histones, that control the dynamic process of spermatogenesis.

Retained histones in mammalian mature sperm contain an array of PTMs, including key genomic regulatory modifications H3K27me3, H3K4me3, and others (Hammoud et al., 2009; Brykczynska et al., 2010; Brunner et al., 2014; Luense et al., 2016). Abnormal sperm histone PTM signatures have been associated with poor sperm quality and infertility, thus further suggesting key regulatory elements of the paternal epigenome. Abnormal, randomly distributed, histone retention was identified in sperm chromatin from infertile men (Hammoud et al., 2011). However, specific genomic localization of H3K4me3 and H3K27me3 was not altered, with the exception of a reduced enrichment at promoters of genes critical for development (Hammoud et al., 2011). H3K9ac was observed at similar levels in sperm from men with normal or impaired spermatogenesis, however differences in the abundance were observed at different genomic loci identified in morphologically abnormal sperm (Steilmann et al., 2011). Reduced total H4 acetylation was observed in human testes with arrested spermatogenesis (Sonnack et al., 2002). Several histone PTMs have been associated with sperm abnormalities, including decreased H4 acetylation in semen samples with asthenoteratozoospermia (abnormal morphology, motility, and forward progression) and asthenozoospermia (abnormal motility and forward progression) (Schon et al., 2019). Abnormal levels of H3K9 and H4K20 methylation were observed in asthenoteratozoospermia and asthenozoospermia samples, but not morphologically abnormal samples alone, suggesting abnormal sperm histone methylation signatures are linked to abnormal motility and progression (Schon et al., 2019). Given the importance of acetylation in proper histone eviction and protamine exchange, as well as transcriptional activation, several downstream ramifications may exist due to a decreased abundance of histone acetylation in sperm. Furthermore, H4K20me is involved in proper DNA replication, DNA damage repair, and in conjunction with H3K9me3, maintain heterochromatic regions that act as a mechanism that results in the transcriptionally quiescent state of mature sperm cells (Schon et al., 2019). These abnormal histone PTM signatures in mammalian sperm linked to infertility and poor embryogenesis suggest additional roles for sperm provided histones to the embryo.

Metabolic state can influence the epigenome through a number of mechanisms, including DNA methylation and sperm-borne small RNAs, however, histone PTMs are particularly susceptible due to their use of common metabolites as substrates for covalent modification (Donkin and Barrès, 2018). Indeed, histone PTM signatures in sperm can be influenced due to diet (Carone et al., 2010). For example, a low-protein diet decreased H3K27me3 at promoters of mitochondrial gene Monoamine oxidase a (Maoa) and elongation factor Tu GTP binding domain containing 1 (Eftud1a), potentially indicating that the sperm epigenome can be regulated by diet-induced changes (Carone et al., 2010). Obesity, a metabolic disease, has also been shown to impact histone PTMs in sperm cells. In mice, H3K18ac, H4K5ac, H4K8ac, H4K12ac, and H4K16ac showed decreased acetylation in genetically obese animals (Fofana et al., 2024). Mice models also demonstrated that male mice fed a folate deficient diet had aberrant chromatin landscapes, specifically in regions of H3K4me3 (Lismer et al., 2021). Promoters with increased H3K4me3 in folate-deficient sperm were enriched for genes associated with early pre- and post-embryogenesis, bone remodeling, and heart development (Lismer et al., 2021). Alternatively, areas of decreased H3K4me3 were enriched for genes associated with osteoclast proliferation, kidney and ear development, and chromatin remodeling (Lismer et al., 2021). Transgenic mice models with overexpression of KDM1a, a histone demethylase enzyme, had enhanced chromatin aberrations when combined with a folate-deficient diet (Lismer et al., 2021). This study indicates the influence of life-long paternal diet on the chromatin landscape of sperm, and the ability of multiple stressors to combine and exacerbate epigenetic effects. Similarly, the use of the KDM1a overexpression model was combined with a high-fat diet to mimic obesity, resulting in differential expression of H3K4me3 at promoters involved in development, cell differentiation, and endocrine and liver function (Pepin et al., 2022). The sperm chromatin profiles of the obese animal model also demonstrated overlap between H3K4me3 regions and genes important for placental and trophectoderm development (Pepin et al., 2022). This indicates that metabolic dysfunction may impact the sperm epigenome at regions of H3K4me3 and may be influenced by methyl donor availability. Furthermore, histone acetyltransferases potentially regulate the metabolic state of the sperm. For example, CBP and P300 responsive genes in elongating spermatids are enriched for carbohydrate metabolism and oxidoreductase activities, thus suggesting regulation of sperm metabolism prior to fertilization (Boussouar et al., 2014). Together, these studies describe a metabolic influence on the epigenetic mechanism of histone PTMs in the sperm.

## Paternal epigenetic contributions to embryo development

Although the genetic contribution of maternal and paternal chromosomes to the embryo is equal, each parental genome carries distinct epigenetic information that is required for embryogenesis. The absence of either parental genome results in embryonic lethality (McGrath and Solter, 1984). The paternal epigenome provides a number of key regulatory features that direct embryonic development, including DNA imprinting, non-coding RNAs, and histone modifications. For example, extraembryonic membranes rely on paternal imprinting of the genome for proper differentiation and development, thus emphasizing the need for paternal chromatin for proper development of the embryo (Surani et al., 1984). Following fertilization, sperm chromatin and the associated epigenome are transmitted to the zygote. Sperm chromatin must rapidly undergo decondensation, as protamines are removed from chromatin and replaced by maternally provided histones (Ecklund and Levine, 1975). Paternally provided histones are retained in embryonic chromatin and remain through the process of syngamy, or the merging of maternal and paternal chromosomes (Van Der Heijden et al., 2008). Recent advancements have identified potential roles for these paternally contributed histones following fertilization that may demonstrate additional importance to their role in spermatogenesis.

Histone PTMs identified in mammalian sperm play potential roles in regulating embryonic chromatin dynamics. Given the conservation of sperm histone PTMs between human and mice (Luense et al., 2016), mouse models have been utilized as a proxy to investigate the role of specific sperm histone PTMs in embryo development. In mouse sperm, the activating histone PTM H3K4me2 is present in sperm at regulatory regions of genes, including transcription start-sites, important for spermatogenesis and cellular processes such as mitosis, DNA repair, transcription, and translation (Brykczynska et al., 2010). Overexpression of the histone demethylase KDM1A during spermatogenesis resulted in decreased H3K4me2 in mature sperm, including at genomic loci important for development (Siklenka et al., 2015). Reduction of H3K4me2 in sperm was observed at two cellular process genes, Pdpk1 and E2F6. Knock-out models of Pdpk1 and loss of E2F6 resulted in profound developmental and skeletal defects in mice, similar to the abnormalities seen in transgenic offspring of mice with altered histone methylation in sperm (Lawlor et al., 2002; Collins et al., 2005; Hashimoto et al., 2006; Courel et al., 2008; Siklenka et al., 2015). Furthermore, major structural and developmental abnormalities as well as decreased survivability were observed for three generations in offspring initially sired from sperm with reduced H3K4me2, suggesting a transgenerational effect (Siklenka et al., 2015). Similarly, H3K4me3, which is typically present at transcriptional start sites, has been localized to genes important for early embryonic development, such as *SOX7, SOX9*, HOX clusters, *FGF9, KLF5, STAT3*, and *ID1*, as well as noncoding RNAs, and paternally expressed imprinted loci in the embryo in mouse and human sperm (Hammoud et al., 2009; Brykczynska et al., 2010). H3K4me3 is maintained in preimplantation embryo development on the paternal allele, escaping reprogramming in the early embryo and indicating a mechanism for transmittance of paternal programming (Lismer et al., 2020). Other histone PTMs that regulate gene transcription at enhancers or promoters, including H3K27ac and H3K9ac (Jung et al., 2017; Bedi et al., 2022), have been identified in mature mouse sperm and further suggest a role of paternal histones poising genes for activation in the early embryo.

The repressive histone PTM H3K27me3 has also been identified in mouse sperm and suggests a critical role in regulation of embryonic gene expression. H3K27me3 is enriched at genes necessary for pattern specification, embryonic morphogenesis, organ development, and nervous system development (Brykczynska et al., 2010). These genes include regulatory genes important for both early and late embryogenesis such as *SOX2, CDX2, GATA6, BMP4, BRACHYURY (T)*, and *HOX* genes (Brykczynska et al., 2010). The Polycomb Repressive Complex 2 (PRC2) mediates H3K27me3 and is comprised of several proteins including EED, EZH2, and SUZ12 (Bracken et al., 2006). Mice homozygous for hypomorphic EED alleles exhibited decreased H3K27me3 in fetal germ cells (Stringer et al., 2018). H3K27me3 enrichment was largely decreased at LINE, LTR, SINE elements, intergenic, and intronic sequences. Because H3K27me3 is generally associated with repressive function, the decrease at these elements correlated to an increase in transcription of LINE elements in the hypomorphic EED mice model (Stringer et al., 2018). These findings further highlight the role of repressive histone modifications regulating genes important for embryo development and indicate a mechanism by which epigenetic information is transmitted through subsequent generations.

Sperm histones also exhibit bivalency, a unique chromatin signature with enrichment of both H3K4me3 and H3K27me3 at the same loci (Hammoud et al., 2009; Brykczynska et al., 2010; Hammoud et al., 2011; Erkek et al., 2013). Bivalent chromatin was first identified in embryonic stem cells and allows for the poising of genes through H3K4me3 enrichment while genes remained repressed (H3K27me3) (Bernstein et al., 2006). Bivalency is resolved during cellular differentiation when H3K27me3 is removed and the presence of H3K4me3 allows for rapid induction of lineage or cell specific gene expression. Bivalent chromatin has been identified in mouse and human sperm at genes critical for developmental and embryonic morphogenesis, including Hox gene clusters (Hammoud et al., 2009; Brykczynska et al., 2010; Hammoud et al., 2011; Erkek et al., 2013). Intriguingly, bivalent chromatin was traced through the male germ cell lineage in mice beginning in primordial germ cells on e12.5 and continuing through meiotic spermatocytes and round spermatids (Lesch et al., 2013), thus suggesting paternally contributed histones result in poising of gene expression throughout the germline. The presence of bivalent chromatin appears to be maintained at a set of genes throughout the germline, thus highlighting the importance of the paternal epigenome in development.

Although transcriptionally quiescent, sperm cells contain both coding and non-coding RNAs. This RNA contributes transcripts to the fertilized oocyte, affecting the viability of the embryo (Boerke et al., 2007). For example, mRNA responsible for translating AKAP-4 plays important roles in both the structural formation of the sperm flagellum, but also in early stages of embryo development, where the protein is required for subsequent signaling in oocyte activation following fertilization (Boerke et al., 2007). In addition to these mRNA transcripts, sperm miRNA contribute to the regulation of gene expression in the developing embryo (Boerke et al., 2007). Small anti-sense RNAs are present in sperm, and remain in the embryo throughout early development (Ostermeier et al., 2005). These RNAs are resistant to degradation and could potentiate a diagnosis for both fertility and future embryo viability. The small RNA epigenetic regulation of the embryo is directly contributed to by the mature sperm, offering yet another potential mechanism by which the paternal genome influences the offspring.

The paternal epigenome is impacted by metabolism during spermatogenesis, which can influence the development of the offspring. Male offspring from mice fed a high-fat diet demonstrated a decrease in sperm H3K9me2 protein content, thus demonstrating intergenerational epigenetic effects from the paternal metabolic state (Claycombe-Larson et al., 2020). Offspring of male mice fed a low-protein diet had depleted H3K27me3 at Maoa promoters in sperm chromatin, and an increase in hepatic expression of genes functioning in lipid and cholesterol biosynthesis (Carone et al., 2010). Male mice fed a diet deficient in folate fathered offspring with higher rates of pregnancy loss, increased incidence of birth defects, and aberrant patterning of H3K4me3 at specific genomic loci (Lismer et al., 2021). Severity of abnormalities in offspring from paternal folate deficiency was exacerbated with the overexpression of KDM1a (Lismer et al., 2021). Promoter regions of aberrant H3K27me3 in folate-deficient sperm overlapped with chromatin abnormalities in the 8-cell embryo, indicating a paternal influence on chromatin in the preimplantation embryo (Lismer et al., 2021). In mice fed a high-fat diet, regions of H3K4me3 in sperm overlapped with open chromatin regions and genes involved in pre-implantation embryo development and placenta development (Pepin et al., 2022). This model indicates that metabolic dysfunction may be mediated by regions of H3K4me3 in sperm and cause generational effects on embryogenesis, chromatin state, as well as placental and trophectoderm function (Pepin et al., 2022). Together, these studies indicate a metabolic influence on the epigenome of sperm cells, particularly on the histone PTMs, and potential roles for paternal environmental factors in future generation’s development.

Our understanding of the role of the paternal epigenome and how it regulates fertility is particularly important as the use of assisted reproductive technologies (ART) become increasingly common. An estimated 15% of couples struggle with infertility (Sharlip et al., 2002), with ∼20% of cases due solely to male factors, and both partners contributing to another 30%–40% of cases (Thonneau et al., 1991). As of 2009, *in vitro* fertilization (IVF), intracytoplasmic sperm injection, and other ART methods have contributed to ∼3 million births since the first IVF baby in 1978 (Cohen, 1978; Hammoud et al., 2009). The percentage of live births generated using ART has continued to increase with 1.9% of newborns conceived by IVF in the US and throughout the world (4.1% in Australia and New Zealand, 5% in European countries, and 1.7% in China) (Bai et al., 2020; De Geyter et al., 2020; Wyns et al., 2020). With the growing use of these technologies, studies have identified a correlation with ART and developmental abnormalities (Hansen et al., 2002; Reefhuis et al., 2009). Disorders appearing in ART offspring include congenital anomalies, low birth weight, prematurity, chromosomal and musculoskeletal defects, and increased incidence of imprinting disorders (Angelmann’s syndrome, and Beckwith-Weldmann’s syndrome) (Cox et al., 2002; Hansen et al., 2002; DeBaun et al., 2003; Gicquel et al., 2003; Ørstavik et al., 2003; Reefhuis et al., 2009; Tararbit et al., 2011). Animal studies have further demonstrated an increase in imprinting disorders following ART, thus supporting the role of epigenetic contributions to the developmental problems in both humans and animals (Maher, 2005; de Waal et al., 2014; de Waal et al., 2015). Furthermore, abnormal sperm chromatin has been linked to recurrent pregnancy loss in humans (Kazerooni et al., 2009; Mohanty et al., 2016; Zidi-Jrah et al., 2016). As ∼50% of ART procedures are performed with sperm that exhibit at least one defect (motility, morphology, integrity, etc.) (Thonneau et al., 1991), a strong possibility exists that subsequent embryos receive an abnormal sperm epigenome. Future research into reproductive epigenetics, particularly on the impact of paternally provided histones on embryo development, will be pivotal in identifying biomarkers, regulatory factors, and novel therapeutics essential for addressing male infertility and ensuring healthy and successful pregnancies.
